# Effect of the application or coating of PGPR-based biostimulant on the growth, yield and nutritional status of maize in Benin

**DOI:** 10.3389/fpls.2022.1064710

**Published:** 2022-12-12

**Authors:** Marcel Yévèdo Adoko, Agossou Damien Pacôme Noumavo, Nadège Adoukè Agbodjato, Olaréwadjou Amogou, Hafiz Adéwalé Salami, Ricardos Mèvognon Aguégué, Nestor Adjovi Ahoyo, Adolphe Adjanohoun, Lamine Baba-Moussa

**Affiliations:** ^1^ Laboratoire de Biologie et Typage de Moléculaire en Microbiologie, Département de Biochimie et de Biologie Cellulaire, Faculté des Sciences et Technique, Université d’Abomey-Calavi, Cotonou, Benin; ^2^ Laboratoire de Microbiologie et de Technologie Alimentaire, Faculté des Sciences et Technique, Université d’Abomey-Calavi, Cotonou, Benin; ^3^ Institut National des Recherches Agricoles du Bénin (INRAB), Cotonou, Benin

**Keywords:** biostimulant, maize, nutritional status, food security, sustainable agriculture

## Abstract

Biotechnology proposes various ecological approaches to control climatic constraints, soil fertility and plant nutrition using biological products, such as biostimulants to achieve a healthy and environment-friendly agriculture. The aim of this study was to compare the effect of biostimulant-coated maize seed and biostimulant application on the growth, yield and nutritional status of maize in Benin. The trials were set up with 100 producers spread over the whole of Benin. The experimental design was a block of three treatments with 11 replicates per Research-Development (R-D) sites. The maize varieties 2000 SYNEE-W BENIN and TZL COMP 4-W BENIN were used. The best growth (height, stem diameter and leaf area) and yield performances (thousand grains weight and grains yield) were obtained by treatments T_2_ (Application of biostimulant + ½ NPK-Urea) and T_3_ (Seed coating with biostimulant + ½ NPK-Urea) compared to the farmers’ practice (T_1_). A significant difference was observed between the different treatments for height, leaf area, 1000 grains weight and maize-grain yield. From one Research-Development site to another, a significant difference was also observed for all parameters. The treatment- Research-Development site interaction was also significant in most areas. The applied or coated biostimulant improved the uptake of nitrogen, phosphorus and especially potassium with higher significant difference compared to the recommended dose of mineral fertilizer. The two techniques of using the biostimulant combined with the half-dose of mineral fertilizer gave the better growth, yield and nutritional status compared to the farmers’ practice in all areas study. This biostimulant can be used to ensure food security and sustainable agriculture in Benin.

## Introduction

Food security is the ability of a population to access and afford enough food to live a healthy life (Olanrewaju et al., 2022). Feeding the growing world population is one of the major challenges for agriculture. In Benin, soils are weakened and subject to deep disturbances, such as physical and chemical degradation, low microbial activity and this has resulted to the decline in the soil fertility with negative consequent to low productivity (Igué et al., 2013). For decades, the application of chemical fertilizers have played a crucial role worldwide to increase growth, crop yield and maintain an adequate food supply (Meena et al., 2017; Chaudhary et al., 2020). Several authors studied the long-term use of chemical fertilizers and their impacts on the agroecosystem (Chaudhary et al., 2017; Bishnoi, 2018). Their applications provide nutrients in high concentrations but with many drawbacks on soil health, water quality and safe environment (Vejan et al., 2016). The use of mineral fertilizers improve productivity, but also leaves undetermined effects on the ecosystem (Kumar and Shastri, 2017). These numerous environmental problems include groundwater pollution, soil acidification, eutrophication, low soil fertility, loss of biodiversity, high energy consumption in synthesis processes, and contamination of crop products (Agbodjato et al., 2016; Tomer et al., 2016; Mahanty et al., 2017; Ilangumaran and Smith, 2017; Kourgialas et al., 2017). To increase crop productivity in an environmentally friendly manner, the use of biostimulants, such as plant growth-promoting microorganisms remain the the most promising ways (De Pascale et al., 2017).

In the last decade, great efforts have been made to substitute chemical fertilizers with environmentally friendly biostimulants (Kamilova et al., 2015; Liu et al., 2018; Thomas and Singh, 2019; Amogou et al., 2021). The response to microbial biostimulants inoculation on different crops has been widely evaluated, thus showing significant increase in the growth and yields of agricultural crops (Babalola et al., 2020; Aguégué et al., 2021b; Adoko et al., 2021a).

Plant growth-promoting microorganisms play an important role in regulating the dynamics of various ecological processes, such as decomposition of organic matter and accessibility of different plant nutrients, such as iron magnesium, nitrogen, phosphorus and potassium (Priyanka et al., 2020). Microbial biostimulants are the main component of integrated nutrient management, thus leading to their sustainability. In addition, these biostimulants can be used as cost-effective biological inputs to increase crop productivity while reducing mineral fertilizer rates and ultimately harvesting more nutrients from the soil (Aguégué et al., 2021a). Biostimulants are sometimes composed of plant growth-promoting rhizobacteria (PGPR), a group of bacteria that actively colonise plant roots, that support plant growth and suppress plant pathogens (Khan et al., 2018). PGPR produce plant hormones (auxin, gibberellin and cytokinin), promote phosphate solubilisation, potassium mineralization and nitrogen fixation, which are important natural organic biostimulants (Rosier et al., 2018). PGPR based biostimulants, with their natures, can play important roles in maintaining soil fertility (Gamez et al., 2019).

Maize is a prevalent crop in Benin’s cropping systems (Gogan et al., 2018). It is very sensitive to low availability of phosphorus and other mineral elements for growth (Postma and Lynch, 2011) and has been shown to be amenable to application of PGPR-based biostimulants (Agbodjato et al., 2021a). Over the last decades in Benin, a lot of work has been done on the use of different biostimulants on maize productivity These studies were carried out on the identification, biochemical characterization, formulation, inoculation and application of PGPR-based biostimulants to increase maize growth and yield (Adjanohoun et al., 2012; Agbodjato et al., 2021b; Adoko et al., 2021b). Nevertheless, studies on the PGPR-based biostimulant-coated maize seed in Benin are less documented. Therefore, the aim of this study was to compare the effect of biostimulant-coated maize seed and biostimulant application on growth, yield and nutritional status of maize in Benin.

## Materials and methods

### Study areas

**Figure 1:**
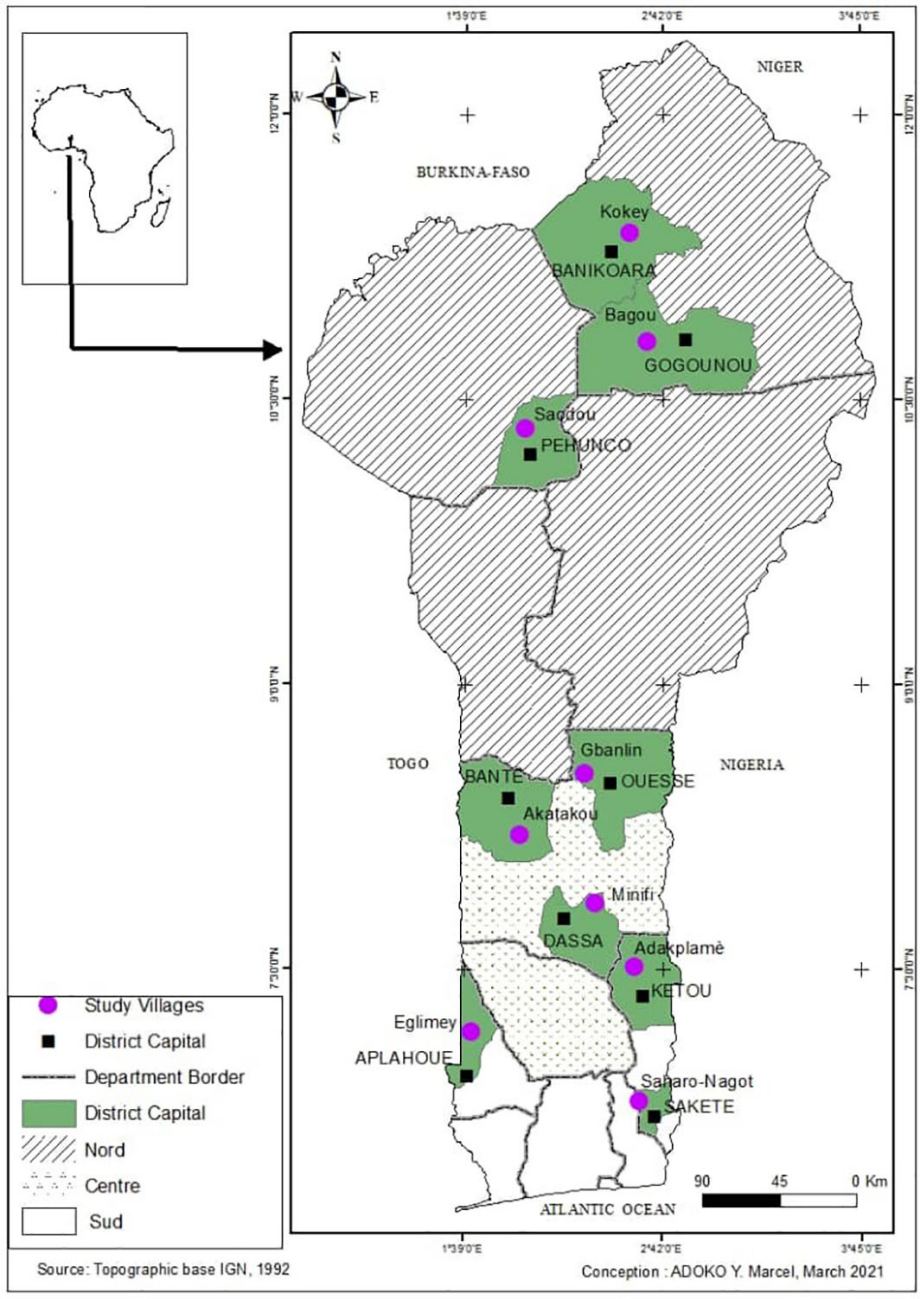
Study areas of the on farmers’ trials in Benin.

Trials were set up at 100 farmers’ sites in different villages of South, Center and North Benin (**Figure 1**). In South with 34 farmers, this includes 11 in Adakplamè (Kétou), 12 in Eglimey (Aplahoué) and 11 in Saharo-Nagot (Sakété). In Center with 33 producers, including 11 in Miniffi (Dassa-Zoumè), 11 in Gbanlin (Ouèssè) and 11 in Akatakou (Bantè). In North with 33 producers, this includes 11 in Soaodou (Péhuncho), 11 in Kokey (Banikoara) and 11 in Badou (Gogounou). Moreover, the selected sites were flat land with a maximum slope of 2%, and not flooded. The decline in the soil fertility was a major constraint. The experimental sites were at least 500 m apart.

### Biostimulant formulation

Method adapted from Connick et al. (1991) was used. Maize flour, bacterial suspension (10^8^ CFU/ml) of *Pseudomonas putida*, binder (Clay) and sucrose were used. These components were put into boxes and mixed with gloved hands until a soft paste was obtained. This was spread on aluminium foil for two days at a temperature of 25°C. After two days of drying, the resulting product was ground in a mortar and then sieved. The different activities were carried out under aseptic conditions. The strain of *P. putida* used in the study was isolated and characterised by Adjanohoun et al. (2011).

### Characterization of maize seed

Maize variety 2000 SYNEE-W BENIN developed by the International Institute of Tropical Agriculture (IITA) and the Institut National des Recherches Agricoles du Bénin (INRAB) was used in the South. It is an extra-early variety of 75 days, with potential grains yield of 2.5t ha^-1^ in a farming environment. In Central and North, the maize variety TZL COMP 4-W BENIN was used. It is a 110-day composite variety with an average grain yield of 4t ha^-1^ on the farm (MAEP, 2016).

### Experimental design

The experimental design was a block of three treatments with 11 replicates per R-D study site where each replicate represented one producer in each site. The treatments were defined as follows: T_1_ = Farmer practice (N_13_P_17_K_17_S_6_B_0,5_Zn_1,5_ (200 Kgha^-1^), Urea (100 Kgha^-1^); T_2_ = Biostimulant application + ½ dose of NPK-Urea (NPK (100 Kgha^-1^), Urea (50 Kgha^-1^); T_3_ = Seed coated with biostimulant + ½ dose of NPK-Urea (NPK (100 Kgha^-1^), Urea (50 Kgha^-1^). Each elementary plot had an area of 40 m² and consisted of five lines of 10 m. The distance between plots was 5 m. The sowing was done at a spacing of 0.80 m x 0.40 m or a density of 31,250 plants ha^-1^ (Yallou et al., 2010).

### Application of the recommended dose of N_13_P_17_K_17_S_6_B_0,5_Zn_1,5_-Urea

The half (½) dose of N_13_P_17_K_17_S_6_B_0,5_Zn_1,5_ was applied as a bottom dressing on the day of sowing on two biostimulants plots. It is only on the farmer’s plot that NPK was applied on the 15th Day After Sowing (DAS). On the other hand, Urea was applied on the 45th DAS for all plots.

### Application of biostimulant and half dose of N_13_P_17_K_17_S_6_B_0,5_Zn_1,5_-Urea

After opening three pits of about 5 cm deep with 2 cm apart, two maize seeds were placed into the central one. Then, 2 g of biostimulant + ½ N_13_P_17_K_17_S_6_B_0,5_Zn_1,5_ were applied separately in the other two pits on the day of sowing. The half dose of Urea was applied on the 45 DAS for all plots.

### Coating of biostimulant and half dose of N_13_P_17_K_17_S_6_B_0,5_Zn_1,5_-Urea

The ratio of 1000 g of seed, 100 g of biostimulant and 100 ml sterile distilled water was used to coat maize seeds. The seeds were mixed and then dried at room temperature for 24 hours. After opening two pits about 5 cm deep and 2 cm apart, two maize seeds coated with biostimulant were placed in one pit and the half dose of N_13_P_17_K_17_S_6_B_0,5_Zn_1,5_ was applied separately in the second pit on the day of sowing. The half dose of Urea was applied on the 45 DAS for all plots.

### Chemical analysis of the soil

Composite soil samples of 500 g were taken at a depth of 0-20 cm from the different sites in the South (Adakplamè, Eglimey, Saharo-Nagot), Center (Akatakou, Gbanlin, Miniffi) and North (Badou, Kokey, Soaodou) prior to the installation of the experimental device using an auger. Five sampling points were randomly selected. Four of the five sampling points were located on the four cardinal points (North-South-West-East). The fifth sampling point was located approximately at the junction of the four previous points (Adjanohoun et al., 2011). These samples were sent to the Laboratoire des Sciences du Sol, Eau et Environnement (LSSEE) of the Institut National des Recherches Agricoles du Bénin (INRAB) for analysis. These analyses consisted of determining the pH (water), (by glass electrode in a soil/water ratio of 1/2.5), organic matter and carbon (Walkley and Black, 1934), assimilable phosphorus (Bray and Kurtz, 1945), total nitrogen (Kjeldahl, 1883), and exchangeable bases by the Metson (1956) method with ammonium acetate at a pH equal to 7.

### Collection of growth and yield data

All growth parameters of the maize plants were measured at 60 DAS in South and 70 DAS in Center and North. Height was measured with a tape meter; stem diameter was measured with a calliper and leaf area was estimated by the product of length and width of leaves with the coefficient 0.75 (Ruget and Bonhomme Chartier, 1996). The ears of 10 maize plants located on central lines of each elementary plot were harvested at 80 DAS in South and at 120 DAS in Center and North. After shelling, total mass of maize grains was measured using a precision balance (Highland HCB 3001. Max: 3000 x 0.1 g) and the moisture content was taken using a moisture meter (LDS-1F). Grains yield was determined according to the formula described by Valdés et al. (2013): R = $\frac{(\text{Px}10.000)\text{ x}14}{(\text{Sx}1.000)\text{xH}}$

where: R = grains yield in t ha-1; P = mass of maize grains in kg; S = harvest area in m²; H = moisture percentage of maize grains in %.

After proper drying until moisture content stabilized (H = 14), the weight of 1000 maize grains were taken according to treatments and R-D sites.

### Assessment of the nutritional status of maize plants

A representative 500 g sample of the dry biomass mixture was taken from each plot. Each sample was placed in a labelled bag. The packaged samples were sent to the Laboratoire des Sciences du Sol Eau et Environement (LSSEE) to determine the N, P, and K content. After dry mineralisation, the ash was dissolved with HCl (6N) and dissolved with HNO_3_ (0.1N). Phosphorus (P) was determined by colorimetry, potassium (K) by atomic absorption spectrophotometry and nitrogen (N) was determined by the method of Kjeldahl (1883).

### Statistical analysis

Effect of experimental area and applied treatments on plant growth and yield performance was assessed by means of a two-factor ANOVA test. Normality and homogeneity of variances of data were performed by ANOVA (Glèlè Kakaï et al., 2006). Thus, Shapiro-Wilk and Levene tests were performed, and type III ANOVA tests were adopted to analyse effect of zone and treatment factors on the parameters evaluated. Once the ANOVA test was significant, a *post hoc* test of pairwise comparisons using Tuckey *post hoc* test (Douglas and Michael, 1991) was carried out to assess statistical differences of the means. In addition, descriptive statistics were calculated per measured parameter. The various tests were performed in the R 4.0.2 software (R Core Team, 2020). These analyses require the use of dplyr and DescTools packages for calculation of descriptive statistics, ggplot2 and ggpur packages for creation of moustache boxes. Stats packages for shapiro test and levene test, the ‘‘car’’ packages for ANOVA and multcomp packages for *post hoc* pairwise comparison test. Significance level was at 5%.

## Results

### Soil chemical characteristics

The soil chemistry characteristics of the R-D sites were presented in **Table 1**. The recorded water pH values (5.78 to 6.80) indicated that the soils were moderately acidic. Cation Exchange Capacity (CEC) values of the soils at the different areas were very low, which ranges from 4.64 to 7.91 cmol^+^ kg^-1^. The available phosphorus was between 4 and 14 mg kg^-1^. The C/N ratio was relatively low and varied between 10.23 and 13.33. The sum of exchangeable bases varied between 3.48 to 9.23 cmol^+^ kg^-1^ (**Table 1**).

**Table 1:** Chemical characteristics of the soils in different Research-Development site.

Research-Development site	Horizons 0 – 20 cm					
	pH water	OM (%)	P-ass (mg Kg^-1^)	C/N	Sum of exchangeable bases (cmol^+^ kg^-1^)	CEC (cmol^+^ kg^-1^)
Eglymè (Aplahoué)	6.30	1.49	9	10.23	9.23	7.91
Adakplamè (Kétou)	6.80	1.57	12	10.43	8	7.82
Saharo-Nagot (Sakété)	6.50	1.50	14	11.12	4.83	5.94
Miniffi (Dassa)	6.20	1.16	4.75	13.33	7.84	8
Gbanlin (Ouèssè)	5.78	1.01	5	12.52	4.22	4.64
Akatakou (Bantè)	5.95	0.55	4	10.80	3.48	6.50
Kokey (Banikoara)	6.30	1.29	5.03	11.88	5.46	5.87
Bagou (Gogounou)	6.08	1.39	5.20	11.57	6.03	7.63
Saodou (Péunko)	6.10	1.36	12.60	12.27	5.92	6.20

pH (water); OM, organic matter; P-ass,assimilable phosphorus; C, carbon; N, Nitrogen; CEC, cation exchange capacity.

### Height of maize plants

**Figure 2:**
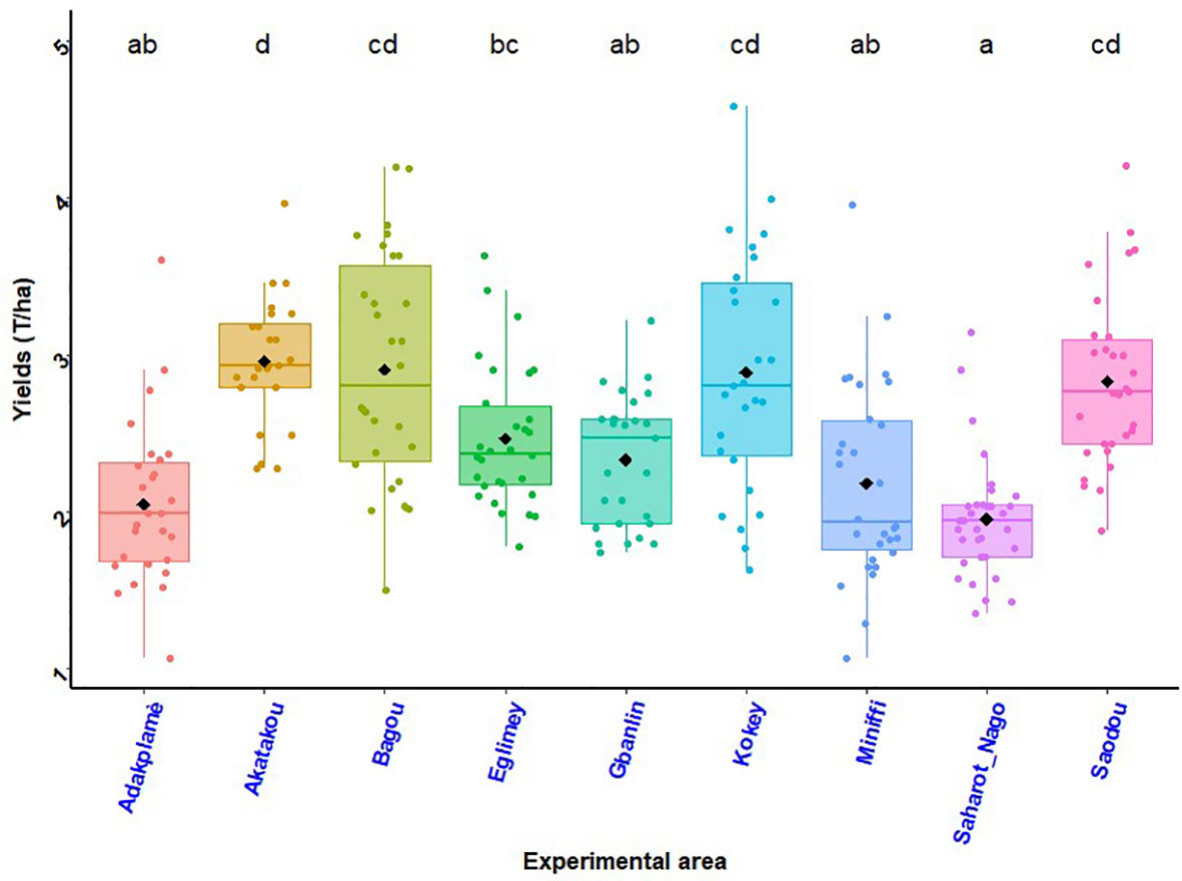
Average height of maize plants per treatment and experimental site. *p* < 0.001 (highly significant). In the same line, means marked with different letters ^i,hi,gi,fgi,efgi etc …^are significantly different at the 5% threshold. T_1_: farmer practice (100% NPK-Urea), T_2_: biostimulant application + ½ NPK-Urea, T_3_: biostimulant coating + ½ NPK-Urea.

The histogram in **Figure 2** illustrates the variation in average height of maize plants per treatment and experimental site. In all R-D study sites in Benin, application of biostimulant + ½ NPK-Urea (T_2_ = 196.57 cm) and the coating of biostimulant + ½ NPK-Urea (T_3_ = 197.20 cm) gave the better performances. A respective increase of 17.65% (T_2_) and 18.03% (T_3_) compared to the farmers’ practice (T_1_ = 167.08 cm) was observed. ANOVA showed a significant difference in the effects of treatments (*p* = 0.001) and experimental sites (*p<* 0.001). Interaction between different treatments and different sites was also significant (*p* = 0.003) (**Figure 2**).

### Stem diameter of maize plants

**Figure 3:**
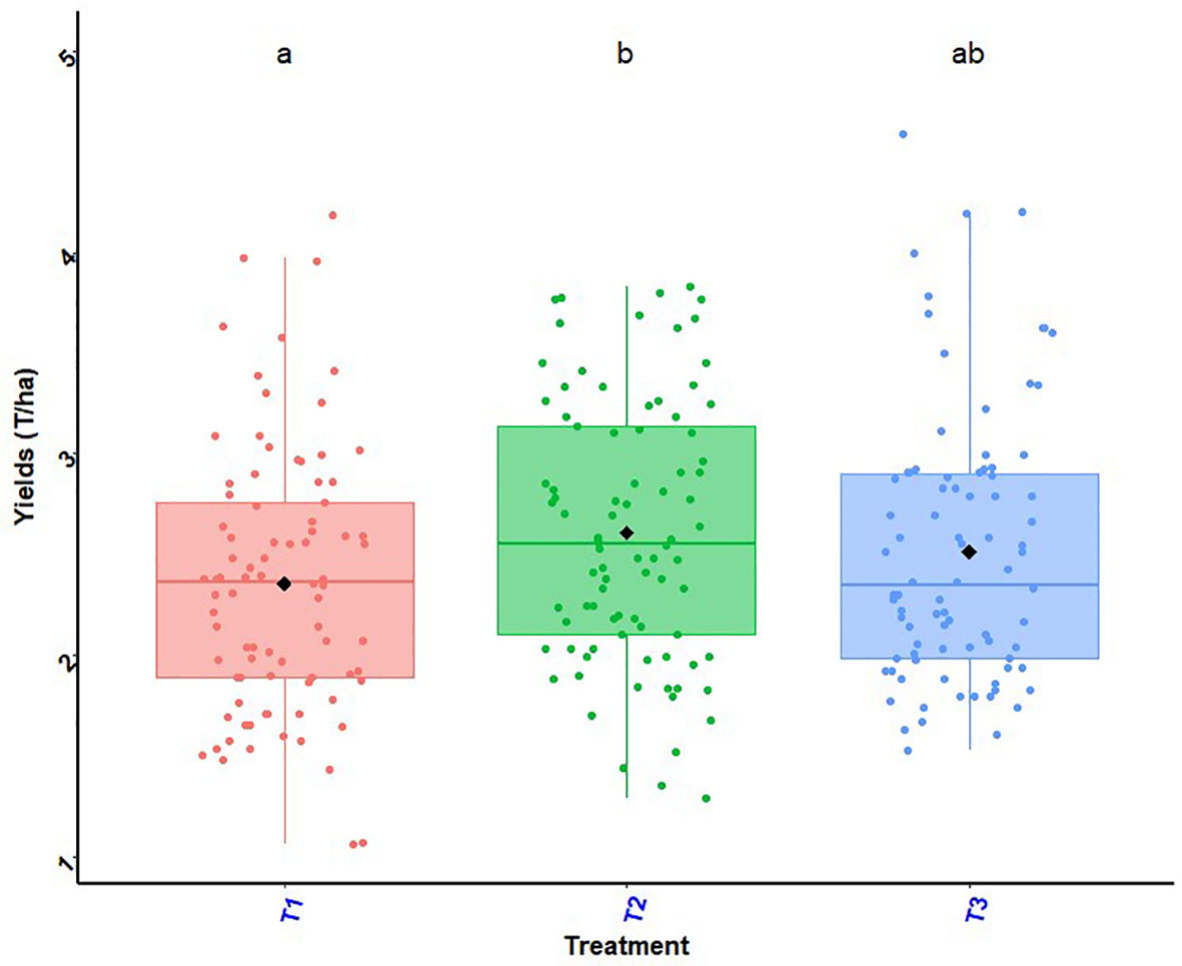
Stem diameter of maize plants by experimental site. *p* < 0.001 (highly significant). In the same line, means marked with different letters ^f,ef,df,de,cd etc …^are significantly different at the 5% threshold.

**Figure 4:**
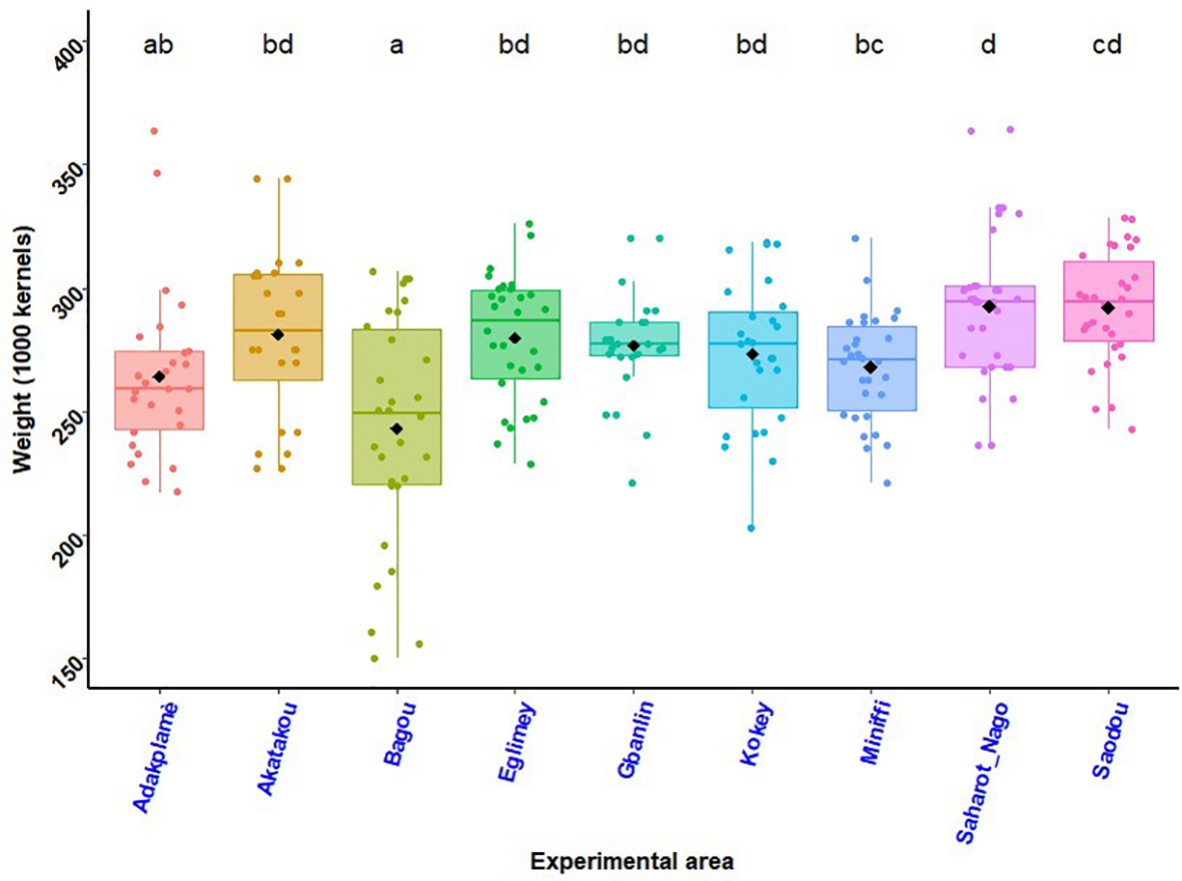
Treatment and site effects on maize stem diameter. *p* > 0.05 (not significant). In the same line, means marked with same letters ^a,a,a^are not significantly different at the 5% threshold. T_1_: farmer practice (100% NPK-Urea), T_2_: biostimulant application + ½ NPK-Urea, T_3_: biostimulant coating + ½ NPK-Urea.

The histogram in **Figure 3** shows the variation in the stem diameter of maize plants as a function of the experimental site and the histogram in **Figure 4** shows Treatment and site effects on maize stem diameter. In all R-D sites, the three treatments had comparable effects on maize stalk diameter (T_2_ = 3.22cm; T_3 =_ 3.17; T_1_ = 3.08cm). The ANOVA revealed no significant difference between treatments in all R-D sites (*P* = 0.09) (**Figure 4**). However, a significant difference in the effects of the experimental site (*p<* 0.001) on the stem diameter of maize plants was found (**Figure 3**). The interaction between treatments and sites was also not significant (*P* = 0.71).

### Leaf area of maize plants

**Figure 5:**
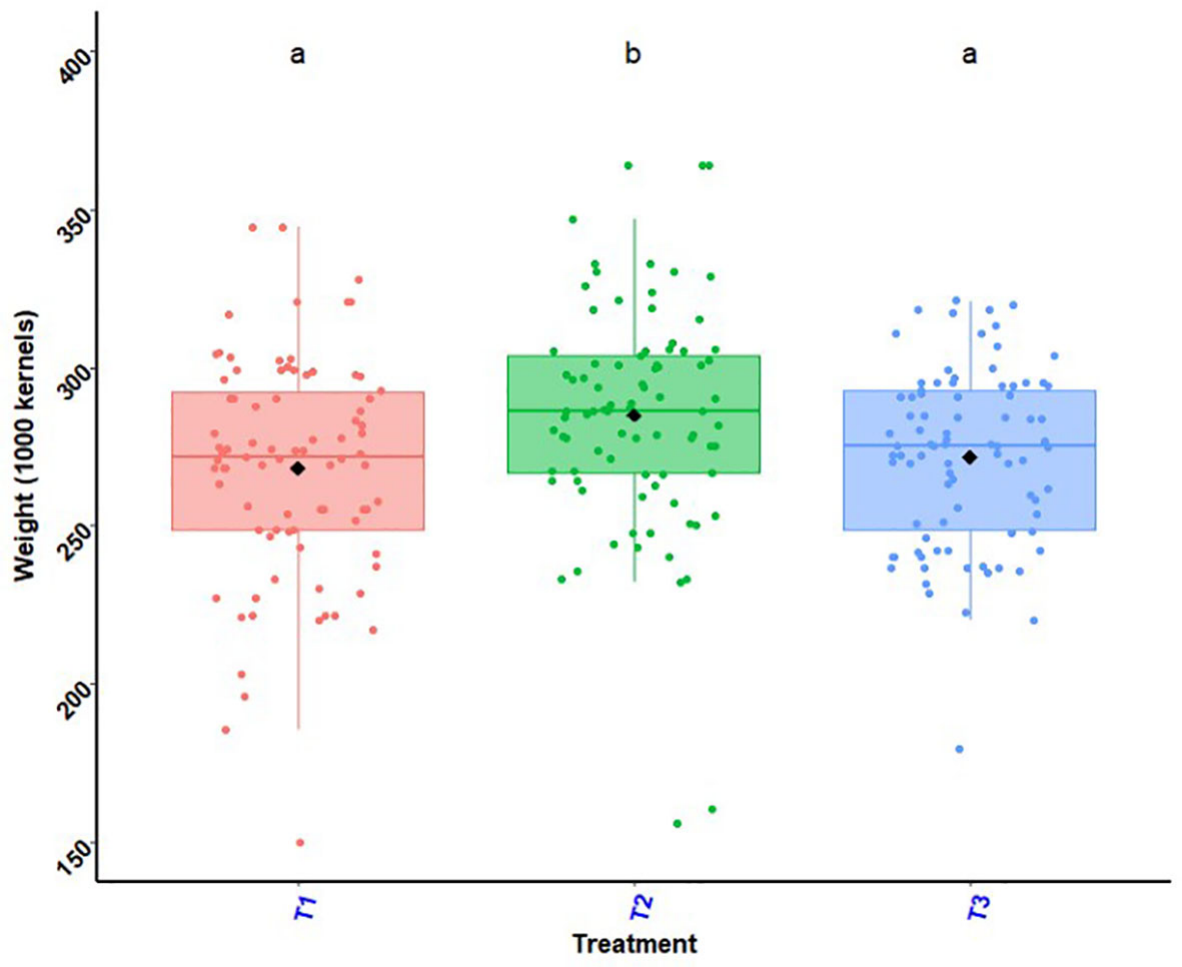
Maize plant leaf area by experimental site. *p*< 0.001 (highly significant). In the same line, means marked with different letters ^e,d,cd,bd etc …^ are significantly different at the 5% threshold.

The effect of biostimulants on leaf area of maize plant by experimental site was illustrated by the histogram in **Figure 5**. Across all study sites in Benin, the largest leaf area (578.36 cm^2^) was obtained in South at Eglimey with farmer practice (T_1_). In Adakplamè, Saharo-Nagot and Soaodou the farmers’ practice and the application of biostimulant + ½ NPK-Urea were better than the seed coating with biostimulant with a significant difference (*p* = 0.015). There was no significant difference between the treatments on leaf area in the R-D sites of Akatakou, Gbanlin, Miniffi, Badou and Kokey. Across experimental sites, the difference was significant (*p*< 0.001). From one experimental site to another, the difference was significant (*p*< 0.001). There was no significant difference between interactions of treatments and the different sites (*p* = 0.061).

### Maize grains yield

**Figure 6:**
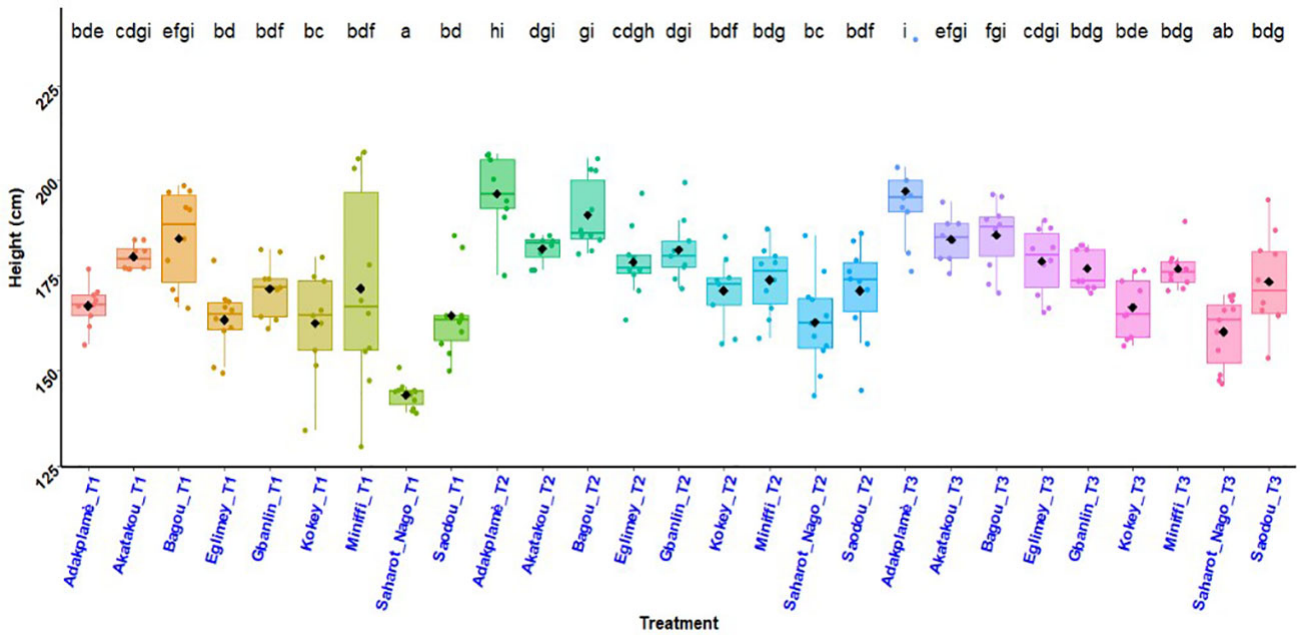
Maize grains yield by experimental site. *p*< 0.001 (highly significant). In the same line, means marked with different letters ^d,cd,bc,ab etc …^ are significantly different at the 5% threshold.

**Figure 7:**
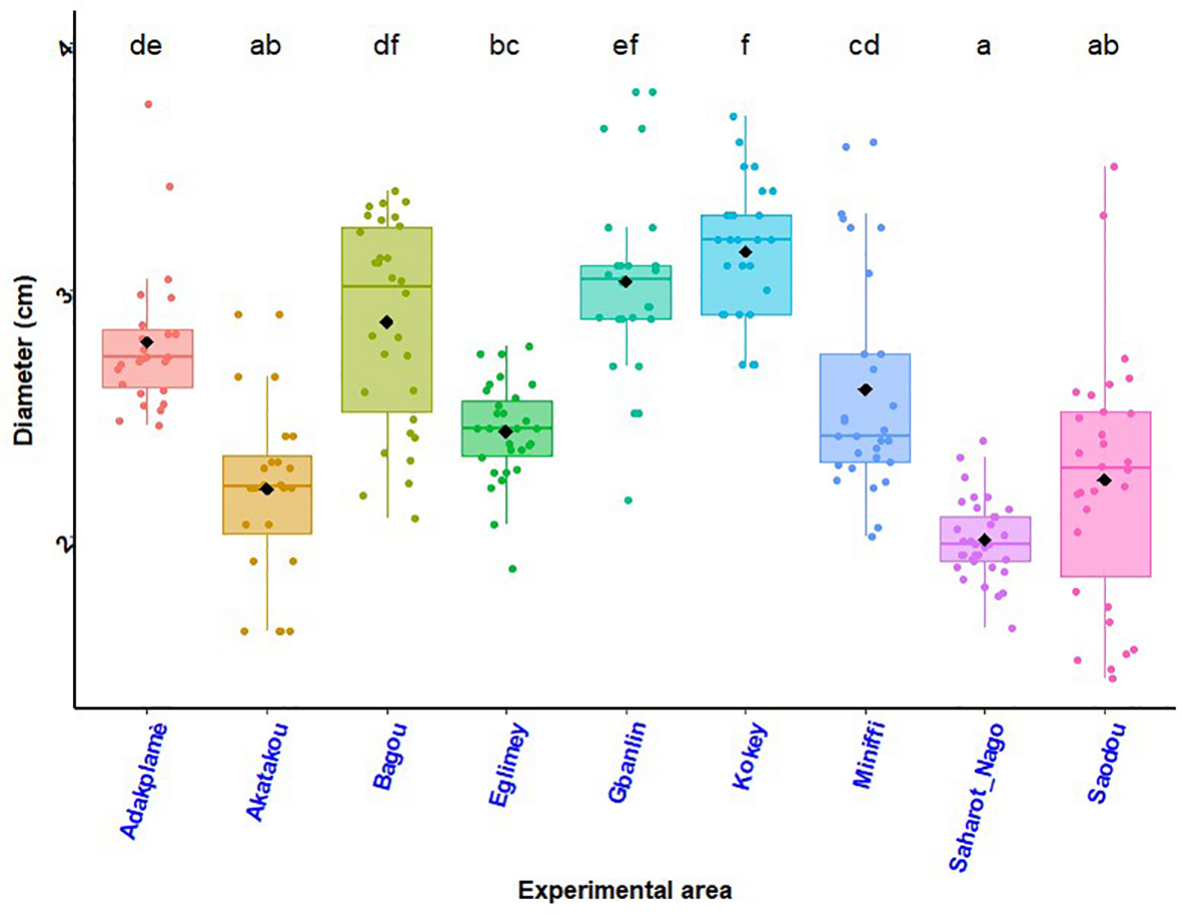
Effects of treatments and site on maize grain yield. *p* < 0.001 (highly significant). In the same line, means marked with different letters ^b,ab,a^ are significantly different at the 5% threshold.T_1_: farmer practice (100% NPK-Urea), T_2_: biostimulant application + ½ NPK-Urea, T_3_: biostimulant coating + ½ NPK-Urea.

Maize grain yields as a function of experimental site illustrated by the histogram in **Figure 6** and the histogram in **Figure 7** shows effects of treatments and site on maize grain yield. The best maize grain yield (T_2_ = 2.48 t ha^-1^) was obtained at Eglimey with the application of biostimulant + ½ NPK-Urea in South Benin. At Adakplamè the application of biostimulant + ½ NPK-Urea (T_2_ = 2.23 t ha^-1^) and seed coating with biostimulant + ½ NPK-Urea (T_3_ = 2.17 t ha^-1^) induced respective increases of 27.42% and 24.00%. In Saharo-Nagot, application of biostimulant + ½ NPK-Urea (T_2_ = 2.10 t ha^-1^) and seed coating with biostimulant + ½ NPK-Urea (T_3_ = 2.00 t ha^-1^) exceeded the farmers’ practice by 20.00% and 14.28%, respectively with a significant difference (*p*< 0.001). In the Centre, the best yield was obtained by the application of biostimulant + ½ NPK-Urea (T_2_ = 3.25 t ha^-1^) at Akatakou. This treatment resulted in an increase of 12.06% compared to farmers’ practice. In Gbanlin and Miniffi, no significant difference was observed between treatments. In the North, the best yield was obtained in Kokey with the application of biostimulant + ½ NPK-Urea (T_2_ = 3.24 t ha^-1^) which had an increase of 31.7%. At Badou, the same treatment was better (T_2_ = 2.99 t ha^-1^) with an increase of 6.03%. In Soaodou, seed coating with biostimulant + ½ NPK-Urea was better (T_3_ = 3.04 t ha^-1^) with an increase of 15.58% compared to the farmers’ practice. The ANOVA results were significant between treatments (*p*< 0.001) and sites (*P* = 0.009) (**Figures 6**, **7**). Interactions between treatments and sites were not significant (*P* = 0.61).

### Maize 1000 grains weights

**Figure 8:**
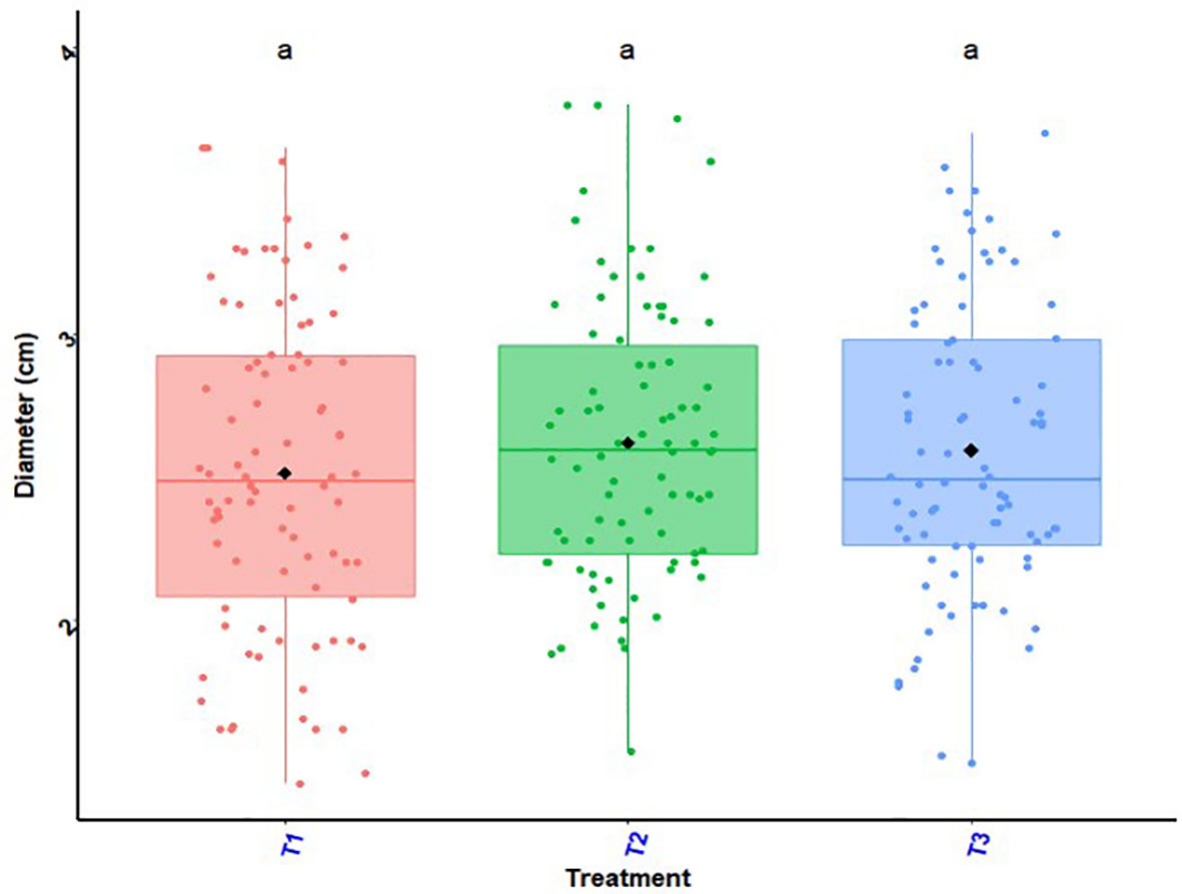
Weight of 1000 maize grains per experimental site.*p* < 0.001 (highly significant). In the same line, means marked with different letters ^d,cd,bd,bc etc …^are significantly different at the 5% threshold.

**Figure 9:**
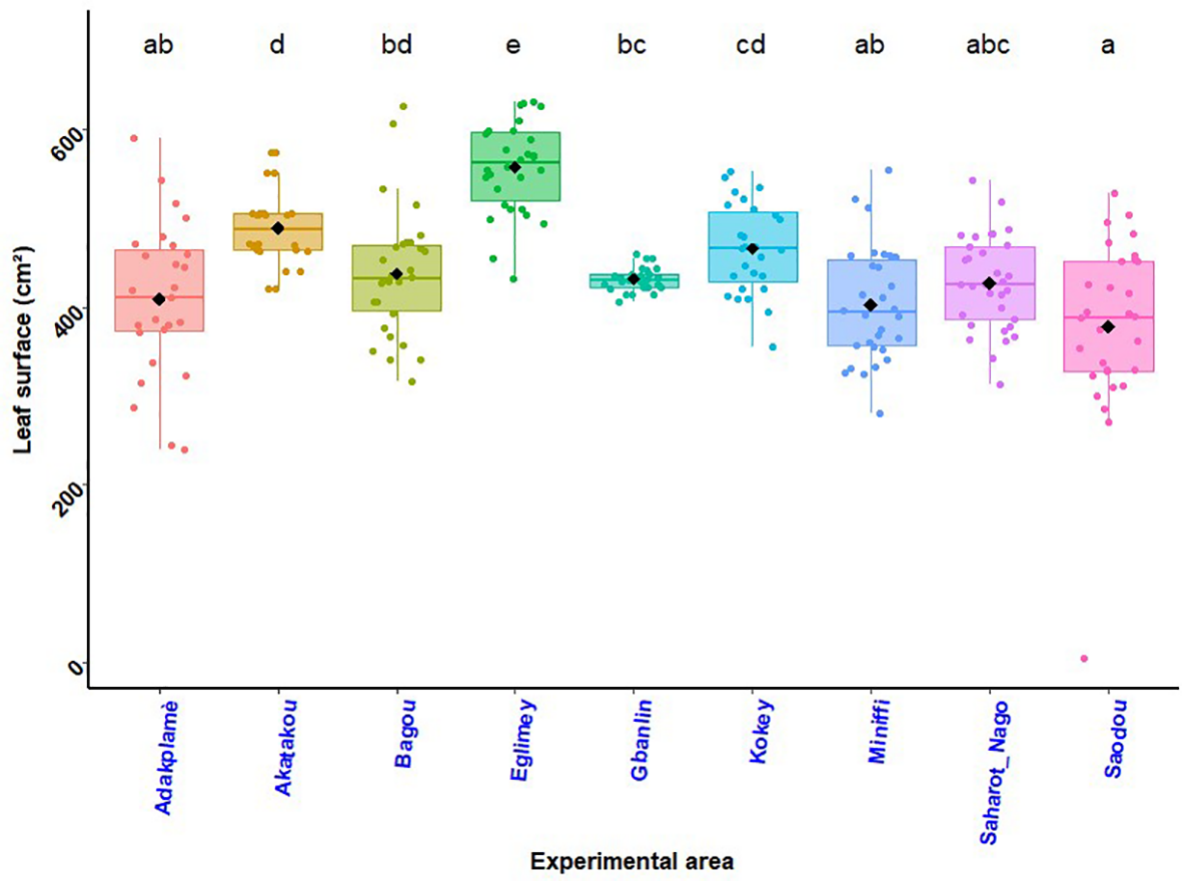
Effects of treatments and site on 1000 grains weights of maize. *p* < 0.001 (highly significant). In the same line, means marked with different letters ^b,a,a^are significantly different at the 5% threshold.T_1_: farmer practice (100% NPK-Urea), T_2_: biostimulant application + ½ NPK-Urea, T_3_: biostimulant coating + ½ NPK-Urea.

Maize 1000 grains weights as a function of experimental site illustrated by the histogram in **Figure 8** and the histogram in **Figure 9** shows effects of treatments and site on 1000 grains weights of maize. In the South, maize grains from the biostimulant + ½ NPK-Urea application had higher weights compared to the other treatments. The highest value of 1000 grains weight was recorded in Saharo-Nagot (T_2_ = 324.60 g) with an increase of 18.44% compared to maize grains harvested with the farmer’s practice. In the Centre, there was no significant difference between treatments. In the North, application of biostimulant + ½ NPK-Urea (T_2_) and seed coating with biostimulant + ½ NPK-Urea (T_3_) were better. In Soaodou, the biostimulant treatments were better than the farmer practice (T_1_ = 285.42 g, T_2_ = 296.02 g and T_3_ = 295.45 g). In all the R-D study sites, variance analysis’s results showed a significant difference between the application of biostimulant + ½ NPK-Urea (T_2_) and the others (T_1_ and T_3_). In addition, there was no significant difference between the farmer’s practice (T_1_) and the seed coating with biostimulant + ½ NPK-Urea (T_3_) (**Figure 9**) Across sites, the difference was also significant (**Figure 8**).

### Nutritional status of maize plants

The nutritional status of maize plants of the R-D sites are presented in **Table 2**. In a whole Benin, nitrogen status did not vary significantly with plants treated with 100% NPK-Urea and with application and coating of biostimulant + ½ NPK-Urea. All inoculated plants improved nitrogen content. For phosphorus, the same thing was observed. Mineral fertilizers, applied and coated biostimulants had practically the same nutritional status. Hence, no significant differences were recorded. The highest potassium levels were obtained with biostimulant + ½ NPK-Urea treatments with significant differences. This was observed in all sites. ANOVA revealed a higher significant difference (*p*< 0.001) between treatments for potassium with no significance for nitrogen and phosphorus.

**Table 2:** Nutritional status of maize plants.

	Traitements	Nitrogen (N)%		Phosphorus (P_2_O_5_)%		Potassium (K_2_O)%	
Zones		M	e	m	e	m	e
	T_1_	1.956^a^	0.162	0.218^a^	0.031	0.389^a^	0.135
South Benin	T_2_	1.897^a^	0.011	0.194^a^	0.088	0.886^a^	0.241
	T_3_	1.873^a^	0.042	0.180^a^	0.083	1.587^b^	0.664
	T_1_	1.864^a^	0.182	0.198^a^	0.071	0.889^a^	0.135
Centre-Bénin	T_2_	1.904^a^	0.111	0.196^a^	0.078	1.486^b^	0.141
	T_3_	1.876^a^	0.142	0.190^a^	0.082	1.587^b^	0.264
	T_1_	1.926^a^	0.162	0.197^a^	0.076	0.489^a^	0.135
North Benin	T_2_	1.987^a^	0.121	0.204^a^	0.068	1.586^b^	0.241
	T_3_	1.983^a^	0.122	0.199^a^	0.086	1.587^b^	0.364
	Significance	ns		ns		***	

***= p< 0.001 (highly significant). ns= p > 0.05 (not significant). In the same column, means marked with different letters ^b,a^are significantly different at the 5% threshold. m: mean. e: standard deviation; T_1_: farmer practice (100% NPK-Urea), T_2_: biostimulant application + ½ NPK-Urea, T_3_: biostimulant coating + ½ NPK-Urea.

## Discussion

The use of microbial biostimulants in agriculture to increase productivity is a natural and environmentally friendly alternative. Results of the initial chemical properties of experimental soils showed that they were slightly acidic. In addition, soils of different R-D sites had a low level of fertility characterised by a high C/N ratio in topsoil. Interaction between different chemical characteristics of soil, which leads to soil fertility classification, reveals that majority of soils in Benin have lost their agricultural potential (Igué et al., 2013). This might be due to very low nitrogen, phosphorus, potassium and cation exchange capacity in the soils. The pH of soil should be neutral to slightly acidic to favour assimilation of mineral elements. Only cation exchange capacity in all soils appears to be a severe to medium limitation regardless of farming system (Igué et al., 2013).

The best performance in height was obtained by coating the seeds with biostimulant, followed by the application of the biostimulant. Statistical analysis revealed in some R-D sites a significant difference in plant height between application and coating of the biostimulant. The difference between the biostimulant treatments can be explained by the seed coating carried out on the day one before sowing to allow a good adhesion of the PGPR contained in the formulation, which can be favourable to the height growth of the maize plants. It makes more PGPR and nutrients available to the plant for better height growth (Neufeld et al., 2017). An increase of 29.43% in the height of wheat plants was observed after the use of the biostimulant *Pseudomonas fluorescens* RRb-11 and talc (Jambhulkar and Sharma, 2013).

In all the study R-D sites, there was no significant difference between treatments on stem diameter of maize plants. However, from one site to another, the differences were significant. The same phenomenon was observed on the leaf area of maize plants in one South R-D site (Eglimey), in all Center R-D sites and in two North R-D sites. In the other two South R-D sites (Adakplamè and Saharo-Nagot) and the North (Soaodou) a significant difference was observed between treatments. These results were related to soil types (Bishnoi, 2015; Adoko et al., 2020; Agbodjato et al.,2021a) and also to maize varieties. Indeed, ferrallitic soils of South had a higher assimilable phosphorus rate (9-14) than ferruginous soils of Center (4-5) and North (5.03-12.6). In South, the 2000 SYNEE-W BENIN variety was used, in Center and North, the TZL COMP 4-W BENIN variety was used during the experiment. Similar results were recorded by Lin et al. (2019) who reported an increase in nitrogen and chlorophyll content throughout the growth stages of the plant that received the biostimulant in combination with mineral fertiliser. The biostimulant applied or coated to the seed makes more nitrogen available to the plant to achieve good photosynthesis for growth. Biostimulants were able to increase yield of cereals. Hassan and Bano (2015) shown that wheat yield was improved by 15-25% over controls with the use of *P. moraviensis* and *B. cereus*.

In all southern R-D sites, the highest maize grains yields were obtained with the application of biostimulant + ½ NPK-Urea, followed by the coating of maize seeds with biostimulant + ½ NPK-Urea. Farming practice gave the lowest yields in all R-D sites. In the majority of these sites, no significant difference was recorded between the application + ½ NPK-Urea and seed coating + ½ NPK-Urea. However, in very few R-D sites, there was a significant difference between the two biostimulant treatments (T_2_ and T_3_) and the farmers’ practice. From one R-D site to another, the differences were significant. The results can be explained by the stimulating effect of a good yield of the rhizobacteria *P. putida* contained in the biostimulant used. This strain was able to produce indole acetic acid (IAA), solubilise certain nutrients, such as phosphate, potassium, make nitrogen (ammonia) and other micronutrients necessary for plant growth and yield enhancement (Noumavo et al., 2015; Agbodjato et al., 2018).

In the Southern R-D sites, maize grains from the application + ½ NPK-Urea had a higher weight than the other treatments. The highest weight of 1000 grains obtained was observed in Saharo-Nagot (324.60 g) with an increase of 18.44% compared to the farmers’ practice. In the Center, there was no significant difference between treatments. In the North, the application + ½ NPK-Urea was also better. The ANOVA showed a significant difference between treatment T_2_ and the others (T_1_ and T_3_) but there was no significant difference between treatments the farmers’ practice (T_1_) and the coating of maize seeds with biostimulant + ½ NPK-Urea (T_3_). Across R-D sites, the differences were also significant. This effect can be due to the soil types, maize varieties and variation in the degree of fertility from one R-D site to another. The amount of biostimulant used for the application (2 g per hole) far exceeds that used for coating two maize seeds (0.1 g). These quantitative differences between application and coating can be the basis of our results. As the amount of biostimulant increases, there was more PGPR and binder (clay) available to the plant, which is favourable for good growth and grains yield and 1000 grains weight.

In all R-D sites, the application and seed coating of biostimulant gave the best results on all parameters (growth, yield and nutritional status) evaluated compared to the farmer practice. This allows us to say that the biostimulant + ½ of NPK-Urea was better than the recommended dose of NPK-Urea for maize cultivation in Benin. This biostimulant provided the plant with nutrients, such as nitrogen, phosphorus and especially potassium (*p*< 0.001) necessary for its growth and yield. Some soil nutrients can also be better absorbed by plants if microorganisms were added. The application of biostimulants was favourable to better increase the biodiversity of the soil microbial community (Ding et al., 2016), which justifies the results obtained in this study.

At some R-D sites in Benin, application and coating of biostimulant had comparable effects. This similarity can be explained by the intrinsic characteristics of *P. putida* known for their roles in promoting crop growth and development. Difference observed was believed to be due to the amount of biostimulant used in the two treatments (Adoko et al., 2021a). Indeed, 2 g of biostimulants applied per hole and less than one twentieth (0.1g) were used in seed coating. PGPR in biostimulants can help the host plant produce root exudates which, in turn, recruit beneficial bacterial communities, thereby increasing dissolved nutrient levels in the soil. Soil organic matter has previously shown to be the main substrate and energy source for microorganisms (Xu et al., 2013). Treatment of wheat seeds with the formulation of *P. fluorescens* RRb-11 in talc resulted in a significant increase in plant growth parameters and even increased the seed germination rate by 94% compared to controls (Jambhulkar and Sharma, 2013). Similarly, Jorjani et al. (2011) reported the efficacy of *P. fluorescens* bioformulation on the fresh weight of sugar beets. Application of inoculated *P. putida* to maize seeds significantly increased the fresh and dry weight of the plants compared to the control at harvest (Khashei et al., 2020). Formulations of talc or peat and *Pseudomonas* spp. effectively controlled chickpea wilt disease in two field trials and increased its yield (Vidhyasekaran and Muthamilan, 1995).

## Conclusion

Agricultural practices that reduce or eliminate chemical inputs through the use of biostimulants have been of paramount importance in recent years for sustainable and environmentally friendly agriculture. The application of biostimulants, such as PGPR in agriculture is one of the promising alternatives to combat abiotic stress and to ensure food security and environmental protection. This study revealed that the biostimulant formulated with *P. putida* and clay binder was effective on growth parameters, yield and nutritional status of maize in Benin. The best treatment was the application of biostimulant + ½ NPK-Urea, followed by the coating of maize seeds with biostimulant + ½ NPK-Urea. Both forms of biostimulant use combined with the recommended half-dose of mineral fertilizer were better than the recommended dose of mineral fertilizer in the majority of the Benin R-D study sites. Biostimulants formulated in Benin can be used to increase maize productivity while reducing the high use of mineral fertilizers. Coating maize seeds with the biostimulant require a small amount of biostimulant, less physical effort than applying the biostimulant, and is better than the recommended practice. However, the formulation process of the biostimulant deserves a slight improvement for a better adhesion of the biostimulant to the seed during coating. Nevertheless, the biostimulant formulated in Benin can be used as a seed coating for sustainable and environmentally friendly agriculture. It will be interesting to continue studies to see the acceptability of the technology by Beninese farmers and to evaluate its financial profitability.

## Data availability statement

The raw data supporting the conclusions of this article will be made available by the authors, without undue reservation.

## Author contributions

MA, AN, NaAA, OA, HS, and R.M.A, carried out the experimental work and analysis. MA, AN, NaAA, A.A., NeAA, and LB-M contributed to the designing, supervision, and interpretation of the results. MA, AN, A.A., NeAA, and LB-M, revised the final draft. LB-M, reviewed the final project. All authors contributed to the article and approved the submitted version.
